# Implementing Food Environment Policies at Scale: What Helps? What Hinders? A Systematic Review of Barriers and Enablers

**DOI:** 10.3390/ijerph181910346

**Published:** 2021-09-30

**Authors:** Binh Nguyen, Leonie Cranney, Bill Bellew, Margaret Thomas

**Affiliations:** Prevention Research Collaboration, Sydney School of Public Health and Charles Perkins Centre, The University of Sydney, Camperdown, NSW 2006, Australia; leonie.cranney@sydney.edu.au (L.C.); william.bellew@sydney.edu.au (B.B.); margaret.thomas@sydney.edu.au (M.T.)

**Keywords:** barriers, enablers, food environment, policy, implementation

## Abstract

Background: Policies that support healthier food environments, including healthy retail food availability and promotion, are an important strategy for obesity prevention. The aim of this systematic review was to examine the evidence for barriers and enablers to successful implementation of healthy food and drink policies, delivered at scale. Methods: MEDLINE, SCOPUS and INFORMIT were searched to May 2019 for peer-reviewed studies. Google and Google Scholar were searched for grey literature. Studies of any design relating to a healthy food and drink policy delivered at scale (≥10 sites) in non-commercial food settings, for specific retail outlets (e.g., vending machines, cafes, cafeterias, school canteens), and that reported on implementation barriers and/or enablers were included. Studies in commercial food retail environments (e.g., supermarkets) were excluded. Studies were appraised for quality and key information was extracted and summarised. Extracted information on barriers and enablers was further grouped into overarching themes relating to perceptions of the policy itself, organisational and contextual factors influencing policy implementation, stakeholder responses to the implemented policy and perceived policy impacts. Results: Of 19 studies, 16 related to policies implemented in schools, two in hospital/health facilities and one in a sport/recreation setting. Most studies were conducted in North America or Australia, and policy implementation occurred mainly at state/regional or federal levels. The most commonly cited barriers across overarching themes and intervention settings were: lack of stakeholder engagement or prioritisation of the policy (11 studies); resistance to change from school stakeholders or customers (8 studies); and concern over profitability, revenue and/or commercial viability (8 studies). Few studies reported on mitigation of barriers. Enablers most commonly raised were: stakeholder engagement, whole-school approach and/or prioritisation of the policy (9 studies); policy level or higher-level support in the form of information, guidance and/or training (5 studies); and leadership, school/policy champion, management commitment and/or organisational capacity (4 studies). Conclusions: Key considerations for policy implementation ranged from building stakeholder support, prioritising policy implementation within organisations, to implementing strategies that address financial concerns and implementation barriers.

## 1. Introduction

Rates of overweight and obesity have grown rapidly in most developed and many developing countries in recent decades [1,2]. The World Health Organisation (WHO) reports that approximately 13% of adults are obese, 39% are overweight, and one-fifth of children around the world are overweight or obese [3]. Overweight and obesity are serious problems for both individuals and nations, resulting in increased risk of morbidity and mortality and poorer quality of life, as well as placing a huge cost burden on health systems and the society overall [4]. It is clear that there are diverse and complex causes of overweight and obesity which require action at multiple levels [5]. Comprehensive policy action by governments is clearly needed to meet WHO targets to reduce obesity and changing food environments has been promulgated as one of the policy interventions most likely to be effective for obesity prevention [3,6,7,8,9].

Food environments are generally defined as the physical, economic, policy, and sociocultural factors that influence people’s choice of food and drinks and nutritional status [9]. Government food policy interventions have the potential to improve the healthiness of food environments by positively influencing food choices, including food purchasing and eating behaviours [10]. While governments have a number of policy options [9,11], food policy interventions that have been implemented in various parts of the world include controls on food advertising, particularly to children, a sugar or sugary drinks tax, nutrient profiling systems such as front-of-pack labelling, and healthy food service policies [12,13,14]. Implementing policies in food environments can influence the characteristics of food and drinks sold within retail outlets such as nutritional quality, availability, and affordability, but also product promotion, placement and point-of-sale nutrition information [15]. Due to the availability of energy-dense and nutrient-poor foods (i.e., unhealthy food products) in many retail food outlets in government-controlled settings, there is a need for large-scale food environment policy interventions that can have an impact on the population [13,16,17,18]. Schools have received much attention as an intervention setting, being a relatively closed environment where children consume a significant part of their daily food intake. There are also opportunities for governments to change healthy retail food availability and promotion in other public institutions and specific settings, such as health facilities, workplaces, sport and recreational settings [8].

Schools have been a focus of food policy interventions for at least the last 20 years [19]. The World Health Organisation (WHO) recently highlighted the importance of healthy school environments in its document ‘Taking Action on Childhood Obesity’, pointing to the opportunity for changing school environments to improve children’s nutrition through providing healthier food and drink options and promoting healthy choices [20]. The WHO recommends standards for meals provided or sold in schools, banning particular products or retail types, and restricting marketing of unhealthy food products within or near schools. In the last two decades, many local and regional healthy school policies, which include requirements for healthy food environments and practices, have been developed and implemented in the United States [21,22], Canada [23], the United Kingdom [24], and Australia [25,26]. In a number of countries, policies to improve food environments have also been implemented in other settings, such as recreational facilities [27,28], hospitals [29,30] and workplaces [31,32,33,34]. There are opportunities to influence food retail outlets within these non-commercial food settings.

However, the implementation of food environment policies in schools or other settings has not always been successful and studies frequently point to the complexity of policy implementation in different contexts and the range of difficulties that hamper implementation [35,36]. A small number of reviews have collated information from policy implementation at both small and large scales and concluded that a strategic approach to policy implementation that recognises and overcomes risks to successful implementation is needed [19]. However, previous systematic reviews have related to policies in the school environment and not extended to other settings [19,37,38], and some have focused on policies based in one country [37,38], a specific time period [38], barriers only [38], and included a mix of small- and large-scale policies [19,37,38].

The aim of this systematic review was to examine the evidence for barriers and enablers to successful implementation of policies designed to increase the availability and promotion of healthy food and drinks (or to decrease the availability and promotion of unhealthy food and drinks) and that have been delivered at scale in food retail environments in various settings. This review explored: (1) settings and countries where government healthy food and drink policies have been implemented at scale and measures used to determine success of implementation; (2) barriers to implementing policies and strategies to mitigate barriers; and (3) enabling factors that support successful implementation of these policies. Findings from this review can help inform the development and implementation of future, large-scale policies seeking to increase the availability and promotion of healthy food and drinks in different non-commercial food retail settings and support healthier food and drink choices in the population.

## 2. Methods

This systematic review was undertaken to inform policy development in NSW [39], and due to time constraints, was not registered on a systematic review protocol registry. Our review complies with the Preferred Reporting Items for Systematic Review and Meta-Analyses (PRISMA) guidelines [40] except where criteria were only applicable to reviews of quantitative studies.

### 2.1. Search Strategy

We developed the search strategy in consultation with a database search specialist and using a PICO framework. The electronic databases MEDLINE, SCOPUS and INFORMIT were systematically searched for relevant studies from the earliest publication date until May 2019, using medical subject headings (MeSH) and keyword search terms. The search strategy was adapted for specific databases. An example of the search strategy used for MEDLINE is presented in Supplementary File S1. Google and Google Scholar were searched for grey literature using the following set of search terms: “healthy food and drink policy implementation barriers enablers”.

### 2.2. Eligibility Criteria and Study Selection

Studies that met inclusion and exclusion criteria summarised in Table 1 were eligible for this review.

**Figure 1:**
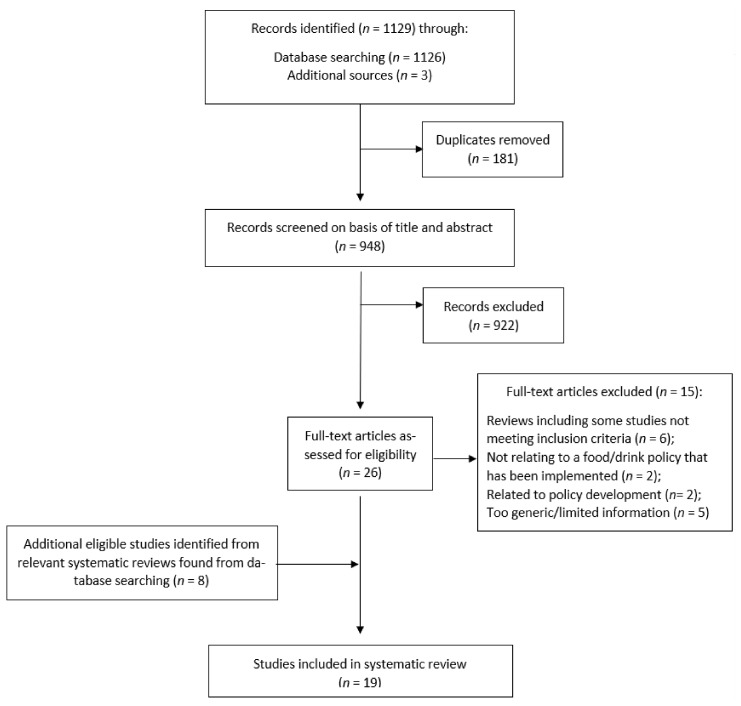
Selection of articles for systematic review.

Articles that were identified through the database and grey literature search were screened for eligibility based on the title and abstract by the first author (BN). Another author (MT) reviewed the abstracts of the articles excluded by the first author to ensure that no relevant articles had been excluded. The full text of potentially eligible articles was screened independently for eligibility by the two authors. Articles that met the inclusion criteria and the consensus of both authors were deemed eligible for review. Additional eligible studies were identified from relevant review articles found during the database search and were subject to the same screening process. A flow chart of studies selected for this review is presented in Figure 1.

### 2.3. Quality Appraisal, Data Extraction and Synthesis

All included studies were appraised for study quality by the first author using the Critical Appraisal Skills Programme (CASP) qualitative checklist [41]. This checklist was appropriate as most included studies were qualitative or included a qualitative component. Each study was critically appraised against 10 individual criteria and given an overall quality rating based on the total number of individual criteria addressed. Studies were rated as “low quality” if less than 4 of 10 individual criteria were met, “moderate quality” if 4–6 of 10 criteria were met and “high quality” if 7 or more of 10 criteria were met.

One author (BN) extracted the following information about each paper: country, policy level (federal, state/regional, other), policy description, target population, setting, scale description, food retail provision type, study design, study description, measurement of implementation success, main findings relating to implementation, type of analysis, stakeholders involved, measurement of implementation barriers and/or enablers, barriers, how barriers were mitigated, enablers, future actions suggested by the paper, and reported study limitations. The information gathered was synthesised into a summary table (Supplementary File S2) with the following information: setting (hospital/health facility, school, sport and recreation, workplaces, other), policy description (level, name, type), whether the policy increases availability/promotion of healthy food and drinks (yes/no), whether the policy decreases the availability/promotion of unhealthy food and drinks (yes/no), scale (large, >25 sites; not so large, ≥10 and ≤25 sites), retail environment, measures of successful/unsuccessful implementation (data collection methods, stakeholders involved, time period), barriers, mitigation of barriers, enablers, and overall study quality rating.

Extracted information in relation to the barriers and enablers to implementing healthy food and drink policies was further grouped into overarching themes relating to perceptions of the policy itself, organisational and contextual factors influencing policy implementation, stakeholder responses to the implemented policy and perceived policy impacts (Supplementary File S3). Organisational factors were defined as factors that occurred within the context of an organisation that implemented a policy, while contextual factors related to factors arising from the context external to the organisation.

## 3. Results

### 3.1. Study Selection

Out of 948 unique records identified, 922 were excluded after screening the titles and abstracts. Following full-text examination of the remaining 26 papers, 19 studies met this review’s inclusion criteria (Figure 1).

### 3.2. Study Characteristics

Study characteristics have been summarised in Supplementary File S2. Seventeen studies (89%) were rated as high quality [24,25,26,28,35,36,42,43,44,45,46,47,48,49,50,51,52] and two (11%) as moderate quality [22,30]. Out of the 15 policies referred to in the 19 studies reviewed, 10 were implemented at a large scale [22,24,25,26,28,30,35,36,42,43,45,47,48,51], nine related to a state/regional policy [22,25,26,28,36,42,43,44,45,46,47,48,50,52], five were a federal policy [24,35,43,49,51], and one was a city-wide policy [30]. Sixteen studies related to implementation of a mandatory policy [22,24,25,26,35,42,43,44,45,46,47,48,49,50,51,52] and three to a voluntarily policy [28,30,36]. Sixteen studies reported both barriers and enablers to implementation [24,25,26,28,30,35,42,43,44,45,46,47,48,50,51,52], while three examined barriers only [22,36,49].

### 3.3. Policy Implementation Settings

Included studies were conducted in six countries, with the highest number of studies stemming from the United States (*n* = 6) [22,30,35,42,50,51], Australia (*n* = 5) [25,26,45,47,48] and Canada (*n* = 5) [24,36,44,46,52]. Sixteen studies related to policies implemented in school settings [22,24,25,26,35,36,42,43,44,46,47,48,49,50,51,52], two in hospital/health facility settings [30,45], and one in a sport and recreation setting [28].

### 3.4. Measures Used to Determine Policy Implementation Success

Four studies used pre–post evaluation designs to determine changes following implementation [22,24,30,46], 13 were cross-sectional studies [25,26,28,35,36,43,44,45,47,48,49,50,51,52] and one was a qualitative study presenting case studies [42]. Definitions of success, which were not always explicitly stated [25], varied among studies and included meeting a defined proportion of specific requirements for the implemented policy or nutrition standards in a given setting [22,24,30,35,44,45]; adoption of guidelines [36]; organisational stage of change in a theoretical model [28] or health framework [46]; perceived improvements in health/quality of foods offered [26]; measured or perceived compliance with the policy [43,47], and perceived outcomes following policy implementation [47,48]. Eleven studies described policy implementation as successful (including the two studies in hospital/health facility settings) [22,24,25,26,30,35,36,45,46,47,48], three reported unsuccessful implementation (two in school settings, one in a sport and recreation setting) [28,43,44] and five did not report on implementation success [42,49,50,51,52].

Most studies used stakeholder surveys (63%), stakeholder interviews (58%) or on-site inventories (21%) to measure implementation. For studies in school settings, stakeholders included mostly school principals, and to a lesser extent, teachers, food service directors, canteen managers, presidents of Parent and Citizens’ associations, and other school staff. For studies in hospital/health facility or sport and recreation settings, stakeholders were most commonly facility managers. Ten studies used mixed methods [24,26,28,36,43,45,46,47,48,51], five used only qualitative methods [42,44,49,50,52] and four used only quantitative methods [22,25,30,35].

### 3.5. Barriers to Policy Implementation

Barriers identified in included studies, according to overarching themes and sub-themes, are presented in Table 2. The three most commonly cited barriers across overarching themes were:

- Lack of stakeholder engagement (e.g., school principals, food service directors, school community) or poor organisational prioritisation of the policy (10 studies in school settings, 1 in a hospital/health facility) [22,35,36,42,43,45,46,48,49,50,51];
- Resistance to change from students, their families and/or canteen staff in school settings [22,24,26,36,43,50,51], and customers in a hospital/health facility setting [45]; and
- Concern from catering/recreational centre managers, school principals and food service directors over profitability, revenue and/or commercial viability (6 studies in school settings, 1 in a hospital/health facility setting, and 1 in a sport and recreation setting) [24,28,36,42,43,44,45,50].

**Table 2:** Barriers identified in included studies according to overarching themes and sub-themes.

Overarching Themes and Sub-Themes for Identified Barriers	Number of Studies	References
Negative perceptions of the policy ^a^	10	[24,28,35,43,44,47,48,50,51,52]
• Misinterpretation/difficulty in understanding policy content/lack of clarity	3	[24,43,44]
• Incompatible/inconsistent with stakeholders’ views on food offerings and consumer demands	3	[28,35,48]
• Nanny state/top-down approach	2	[24,50]
• Reduced parental autonomy	2	[47,51]
• Too restrictive	1	[52]
Implementation factors	15	[22,24,25,30,35,36,42,43,45,46,48,49,50,51,52]
*Organisational*	12	[22,30,35,36,42,43,45,46,48,49,50,51]
• Lack of stakeholder engagement, prioritisation of the policy	11	[22,35,36,42,43,45,46,48,49,50,51]
• Lack of time, money, staff, resources	6	[30,35,43,46,49,51]
• Lack of leadership, management commitment	2	[35,45]
• Ineffective implementation processes adopted	1	[43]
*Contextual*	15	[22,24,25,30,35,36,42,43,45,46,48,49,50,51,52]
• Lack of supply of policy-compliant/healthy products	5	[24,30,35,42,45]
• Rural facility location (vs. urban), school type (e.g., primary vs. secondary, public vs. private), non-supportive management structures, external management of food supply	5	[24,25,30,36,43]
• Lack of information, guidance and/or training support from the policy level	5	[35,36,43,49,52]
• Lack of enforcement	3	[35,43,49]
• Difficulty forming partnerships/conflicts of interest	2	[46,49]
• Marketing and promotion of EDNP foods within facility	1	[43]
Stakeholder responses ^b^	10	[22,24,26,28,36,43,44,45,50,51]
• Consumer resistance (e.g., personal preferences, family habits)	8	[22,24,26,36,43,45,50,51]
• Complaints (e.g., educators overstepping boundaries/undermining parental authority, less convenient)	5	[26,28,44,45,51]
• Food/drink purchase displacement externally	2	[24,28]
Perceived policy impacts	11	[22,24,26,28,36,42,43,44,45,50,52]
• Loss of profits/revenue, commercial viability	8	[24,28,36,42,43,44,45,50]
• Higher food cost/food insecurity	5	[22,26,36,44,52]
• Food/drink external displacement due to access to external food outlets ^c^	2	[43,52]
• Increased labour cost	1	[22]
• Difficulty finding fundraising alternatives	1	[44]

Abbreviations: EDNP, energy-dense nutrient-poor. ^a^ Perceptions from parents, principals, teachers, canteen managers, Parent and Citizens’ associations representatives. ^b^ Stakeholders included school principals, teachers, staff, catering/facility managers, food and service directors/supervisors. ^c^ Perceptions of school principals/staff.

#### 3.5.1. Perceptions of the Implemented Policy

Ten studies (nine in school settings, one in a sport and recreation setting) reported negative perceptions of the implemented policy [24,28,35,43,44,47,48,50,51,52]. Difficulty understanding the policy [24,43,44] and the policy not aligning with stakeholders’ views or demands (i.e., parents and students, consumers) in terms of foods and drinks offered were commonly reported as negative perceptions [28,35,48].

#### 3.5.2. Organisational and Contextual Factors Influencing Policy Implementation

- Twelve studies (10 in school settings, two in hospital/health facility settings) mentioned organisational barriers to policy implementation [22,30,35,36,42,43,46,48,49,50,51], most frequently lack of engagement and/or prioritisation of the policy by stakeholders (e.g., school principals and staff, parents) [22,35,36,42,43,45,46,48,49,50,51], and lack of time, money, staff and/or resources [30,35,43,46,49,51].
- Fifteen studies (13 in school settings, two in hospital/health facility settings) reported contextual factors as barriers to policy implementation [22,24,25,30,35,36,42,43,45,46,48,49,50,51,52]. Commonly cited barriers were lack of supply of policy-compliant or healthy products [24,30,35,42,45], school location, type, and/or management structures [24,25,30,36,43], and lack of information, guidance and/or training support from the policy level [35,36,43,49,52].

#### 3.5.3. Stakeholder Responses to the Implemented Policy

Ten studies (eight in school settings, one in a sport and recreation setting, one in a hospital/health facility setting) reported negative stakeholder responses as barriers to policy implementation [22,24,26,28,36,43,44,45,50,51]. Surveyed or interviewed stakeholders were mostly school principals [26,36,43,44,50,51], followed by school teachers/staff [22,36,44], catering/facility managers [24,28,45] and food service directors/supervisors [22,50]. Study participants most frequently reported the following responses as barriers, generally those of other stakeholders affected by the implemented policy:

- Resistance to change from students, their families and/or canteen staff in school settings [22,24,26,36,43,50,51], and customers in a hospital/health facility setting [45];
- Complaints from parents (e.g., role of educators, parental authority being undermined) [26,44,51] and from managers of recreational facilities (e.g., lower convenience of preparing/storing healthy foods) [28] and of health facilities (e.g., lack of demand) [45].

#### 3.5.4. Perceived Policy Impacts

Eleven studies (nine in school settings, one in a hospital/health facility setting, one in a sport and recreation setting) reported perceived negative impacts of policy implementation [22,24,26,28,36,42,43,44,45,50,52], most frequently relating to principals’, food service directors’ or outlet managers’ concerns about financial impacts or commercial viability [24,28,36,42,43,44,45,50], and concern over cost of healthy foods and the potential impact on food security for vulnerable students [22,26,36,44,52].

#### 3.5.5. Barriers Reported in Studies Reporting Successful versus Unsuccessful Implementation

The three studies reporting less successful policy implementation [28,43,44] all cited negative perceptions of the policy, and concern over loss of profits, revenue and/or commercial viability as barriers. Barriers from all four overarching themes were mentioned in studies reporting successful implementation.

#### 3.5.6. Barriers Reported in Studies Relating to a Mandatory versus Voluntary Policy Implementation

Studies relating to a policy implemented voluntarily cited organisational and contextual factors [30,36], negative stakeholder responses [28,36], and concern over profitability or commercial viability [28,36] as barriers. Barriers from all four overarching themes were mentioned in studies relating to a mandatory policy implementation.

### 3.6. Mitigation of Barriers

Only three studies described how barriers were mitigated, and reported the following strategies:

- Increasing stakeholder engagement by involving community members in discussions and supporting existing partnerships with external organisations, for example schools working with local health organisations to host health-promoting activities as part of a “wellness week” (school setting) [46];
- Taking a long-term approach to help students and vending machine suppliers to adapt to changes with time (school setting) [42];
- Non-negotiable and permanent nature of a school policy helping to settle complaints from parents and children (school setting) [26]; and
- Training of school canteen staff to develop canteen menus that comply with the policy and that consider infrastructure and staffing constraints (school setting) [26].

### 3.7. Enabling Factors

Enablers identified in included studies according to overarching themes and sub-themes are summarised in Table 3. The enabling factors most commonly raised across overarching themes were:

- Stakeholder engagement, whole-school approach and/or prioritisation (nine studies in school settings) [24,25,26,35,42,43,47,51,52];
- Policy level or higher-level support in the form of information, guidance and/or training (five studies in school settings, one in a hospital/health facility setting) [26,30,44,45,46,47]; and
- Leadership, school/policy champion, management commitment and/or organisational capacity (four studies in school settings, one in a sport and recreation setting) [28,35,42,46,52].

**Table 3:** Enablers identified in included studies according to overarching themes and sub-themes.

Overarching Themes and Sub-Themes for Identified Enablers	Number of Studies	References
Positive perceptions of the policy	4	[25,44,47,48]
• Easy to understand	4	[25,44,47,48]
• In line with stakeholders’ views/demands	2	[47,48]
• Nanny state/top-down approach	1	[44]
• In line with parental rights	1	[47]
Implementation factors	13	[24,25,26,28,30,42,43,44,45,46,47,51,52]
*Organisational*	11	[24,25,26,28,35,42,43,46,47,51,52]
• Stakeholder engagement, whole-school approach, prioritisation	9	[24,25,26,35,42,43,47,51,52]
• Leadership, school/policy champion, management commitment, organisational capacity	5	[28,35,42,46,52]
• Effective implementation processes adopted	1	[42]
*Contextual*	9	[24,26,30,42,43,44,45,46,47]
• Information, guidance and/or training support from the policy level/higher-level support	6	[26,30,44,45,46,47]
• Supply of policy-compliant/healthy products	2	[24,44]
• Healthy eating marketing	1	[42]
• Previous involvement with a voluntary food categorisation system	1	[47]
• Monitoring/enforcement of policy compliance	1	[43]
• External partnerships with the community (e.g., local farms, community centres)	1	[42]
• Part of a multisector effort	1	[30]
Stakeholder responses	6	[26,30,44,47,48,50]
• Acceptance of policy/change, positive attitude	4	[26,44,47,50]
• Ease of implementation, policy providing legitimacy to make changes	2	[26,48]
• Public recognition of accomplishments	1	[30]
Perceived impacts	2	[25,48]
• Belief in profits/revenue, commercial viability	1	[25]
• Increased availability of healthy foods	1	[48]

#### 3.7.1. Perceptions of the Implemented Policy

Four studies in school settings mentioned positive perceptions of the policy as enabling factors [25,44,47,48]. All studies referred to ease in understanding the policy, three reported a good understanding of the food and drink classification system [25,47,48], and two mentioned the policy reflecting school stakeholders’ views [47,48].

#### 3.7.2. Organisational and Contextual Factors Enabling Policy Implementation

Eleven studies (10 in school settings, one in a sport and recreation setting) cited organisational factors as enablers [24,25,26,28,35,42,43,46,47,51,52], most commonly:

- Stakeholder engagement, prioritisation and/or a coordinated approach across the school [24,25,26,35,42,43,47,51,52].
- Leadership, policy champion, management commitment and/or organisational capacity such as putting in place administrative procedures and a task force committee to implement the policy [28,35,42,46,52].
- Enabling contextual factors were also reported by nine studies (seven in school settings, two in hospital/health facility settings) [24,26,30,42,43,44,45,46,47]. The most frequently cited factors were access to information, guidance and/or training support such as implementation guides, support materials, technical assistance, or training of canteen managers, from the policy level or higher-level support [26,30,44,45,46,47], and availability of policy-compliant or healthy products from suppliers [24,44].

#### 3.7.3. Stakeholder Responses to the Implemented Policy

Six studies in school settings reported positive stakeholder responses as enabling factors [26,30,44,47,48,50]. The most common responses were stakeholder’s acceptance of or positive attitudes towards the policy [26,44,47,50], ease of implementation, and providing the authority and justification for schools to make changes [26,48].

#### 3.7.4. Perceived Policy Impacts

Two studies in school settings cited perceived positive impacts of policy implementation [25,48]. Dick and colleagues found that most Parent and Citizens’ associations reported believing in the financial viability of healthy school food shops (78%) and healthy fundraising (62%), and 56% reported increased (15%) or unchanged (41%) school food shop profits [25]. In a study about the implementation of a healthy food and drink policy in schools in Western Australia, most stakeholders agreed that the policy had been effective in making healthier food offerings in schools one year (84%) and 10 years (85%) after implementation [48].

#### 3.7.5. Enablers Reported in Studies Reporting Successful versus Unsuccessful Implementation

Studies reporting unsuccessful policy implementation [28,43,44] only cited enablers relating to perceptions of the policy [44], organisational and contextual factors [28,43,44], and stakeholder responses [44]. Enablers from all four overarching themes were mentioned in studies reporting successful implementation.

#### 3.7.6. Enablers Reported in Studies Relating to Mandatory versus Voluntary Implementation

Two out of three studies relating to voluntary policy implementation [28,30,36] reported enablers, including having a policy champion [28], support from high-level officials or leaders, information, guidance and/or training support from the policy level, and public recognition of accomplishments [30]. Enablers from all four overarching themes were mentioned in studies relating to mandatory implementation.

## 4. Discussion

This review, based on 19, mostly high-quality eligible studies, identified key barriers and enablers to the implementation of healthy food and drink policies at scale. Most of the evidence came from studies conducted in the school setting and provides substantial information about implementation barriers and enablers in schools, especially in North America. This evidence, however, may not be generalisable to other intervention settings or countries where the school system differs from that of North America. Findings from the limited number of studies (*n* = 3) conducted in hospital/health facility and recreational settings may serve as preliminary evidence.

The review findings indicate food policy implementation at scale has occurred less frequently in settings other than schools, as confirmed by other reviews [53]. To some extent, this reflects the relative ease of implementation of food environment policies at scale in schools compared to other more dispersed and less controlled environments. There were, however, commonly cited factors that impacted on successful policy implementation across all intervention settings in this review, including several organisational and contextual factors relating to stakeholder response and influence, organisational leadership, and management and support. Stakeholders are clearly central to successful policy implementation because if they understand what the policy is trying to achieve and openly support rather than resist the changes, policy implementation will be enhanced. Our findings echo those of a recent evidence synthesis and consensus report which found that stakeholder engagement and support are central to healthy food procurement policy development and implementation [53]. Engaging all stakeholders early and communicating effectively with them throughout the implementation process seems essential to facilitating policy implementation.

Introducing policy changes also requires organisational leadership and good management. Receiving the guidance and support needed at the organisational level can help stakeholders to increase their understanding and support of the policy, adapt and comply with implemented changes, and facilitate the implementation process. Good leadership at the policy level can also provide background support for implementation within organisations through policy prioritisation and commitment. Overall, effective communication at all levels and providing the necessary organisational and policy level support for the changes being implemented will help to enhance policy implementation.

Providing the necessary types of support, including addressing practical issues such as product availability, need for information and technical assistance, and providing training, will enable implementation through reducing stakeholder resistance. Implementers need to be aware of the changing attitudes and expectations of stakeholders and consumers, including resistance and dissatisfaction, and respond appropriately. The impact on implementation of known or unexpected contextual factors which can arise during implementation need to be successfully managed.

Given that implemented policies related to food retail environments, it is not surprising that potential revenue losses and profitability were key concerns that tended to impact negatively on implementation. Considering strategies to address financial concerns of stakeholders and maintain profits of food retail outlets may help to mitigate this important barrier and favourably influence stakeholders’ perceived financial impacts of the policy. A systematic scoping review examining business outcomes of healthy food retail strategies recognised the importance of a holistic approach that takes into account commercial viability, customers’ perceptions, but also retailers’ perspectives and broader community outcomes [54].

The overarching themes for both barriers and enablers, namely stakeholder perceptions of the implemented policy, organisational and contextual factors influencing policy implementation, stakeholder responses to the policy and stakeholder’s perceived policy impacts, are linked and mutually reinforcing of each other and the implementation process. For example, if the policy and implementing strategies are not well communicated to stakeholders, the implemented policy may not be well perceived by stakeholders and they are less likely to be engaged in implementation delivery within the organisation. Poor stakeholder engagement at early stages of implementation and insufficient resources supporting stakeholders at sites where the policy is being implemented will result in a further lack of stakeholder engagement and may lead to poorer organisational implementation. These unfavourable factors can influence stakeholder responses, such as resistance to change, and negative perceived impacts of the implemented policy.

Outside of school settings, there was a limited number of barriers identified and these were a sub-set of those identified in schools. Healthy food and drink policies delivered at scale in settings such as hospitals, health facilities, and sport and recreation centres, which are open to both children and adult populations, may be challenging to implement successfully due to the number and various types of commercial food retail outlets available. These settings may provide a different context to schools in terms of organisational goals, culture and consumers targeted, and there could be other, as yet unidentified, barriers and enablers impacting implementation. In order to identify any unique barriers and enablers in other settings, including workplaces, studies using comprehensive or robust evaluation designs including mixed methods are needed. This would provide evidence for policy makers and other stakeholders seeking to increase the availability and promotion of healthy food and drinks in these specific settings.

Of interest is that few studies specifically examined or reported on strategies used to mitigate barriers. This additional information is needed given the importance of understanding how mitigating barriers could lead to more successful implementation; research and evaluation should focus more on this aspect of policy implementation. In recent years, co-production between public health practitioners or researchers, and stakeholders or intervention providers, has re-emerged as an important principle in designing public health interventions and research [55,56,57]. The benefits of co-production and diverse stakeholder engagement are that they provide valuable understanding of implementation context and relevance, which enhances the ability to anticipate and mitigate barriers and the potential for implementation success. Another gap found in this review was the limited reporting on implementation success, so that the impact of the identified barriers and enablers was difficult to assess. Further studies that clearly define, measure and report on implementation success would be valuable.

One of the strengths of this review is that it incorporated all types of study designs, and in comparison to previous reviews [19,37,38], extended to all settings, countries, time periods, and examined both barriers and enablers of policies implemented at scale. A refined search strategy and careful processes of review were used to identify relevant studies that met the inclusion criteria. Included studies were reviewed in detail, ensuring that the most relevant information was extracted. We acknowledge that our review only included food policy implementation studies at scale that reported on barriers and enablers, so more information is available in the literature about the impact of these at scale food environment policy interventions, as well as the enablers and barriers of smaller-scale food retail environment interventions in various settings which were not included in this review [19,27,28]. The findings of this review, however, were comparable to those of a recent systematic review involving smaller-scale food retail environment interventions [58]. Similar barriers were reported including the need for stakeholder engagement, consumer demands, limited healthy product availability, and perceived business costs and commercial viability [58].

This systematic review used systematic searches that aimed to be comprehensive. However, due to time constraints, it is possible that the searches undertaken may have missed relevant studies for this review. In addition, findings were based on studies conducted mainly in three countries and may not be generalisable to other countries. While a quality assessment was undertaken using a reputable assessment tool, data collected using qualitative methods may be subject to interviewee bias. Data collected quantitatively may also be subject to self-report bias but there was consistency between quantitatively and qualitatively collected data in studies where both were collected indicating that triangulation of data sources produced similar results. The few studies using pre–post designs, and lack of studies using quasi-experimental, or controlled designs clearly limits the quality of the evidence. Confidence in the findings from studies in health facilities or recreational settings in this review is limited because of the low number of studies available.

This review revealed a common set of key barriers and enablers to implementation of food environment policies at scale and should provide valuable information for food policy implementers. Further studies are needed to determine the feasibility of implementing healthy food and drink policies implemented at scale across various settings and to understand their impacts on stakeholders. Studies from various countries with strong evaluation designs, sound measures of implementation success, and information on costs/profits of retail outlets following implementation changes and on successful mitigation of barriers would add significantly to the body of knowledge on food environment policy implementation at scale.

## Figures and Tables

**Table 1:** Inclusion and exclusion criteria.

Items	Inclusion Criteria	Exclusion Criteria
Study type	Studies of any design	None
Policy description	All types of healthy food and/or drink policies * implemented on a scale that can have an impact at a broad community level Include federal, state/regional policies	Not related to implementation of a healthy food and/or drink policy Unlikely to have a broad community level impact
Scale	At scale (≥10 sites **)	Scale too small (<10 sites)
Settings	Non-commercial *** food retail settings (government or non-government) Hospitals/Health services Schools Sport and recreation Workplaces Museums Zoos Stadia	Commercial food retail settings Food service (e.g., in-patient hospital food, government-subsidised school meals) Childcare settings
Type of food retail provision	Vending machines Workplace cafeterias Cafes Kiosks School canteens Fundraising outlets	Shopping centres Supermarkets Convenience stores Catering services One-off fundraising outlets (e.g., school bake sales)
Promotional activities	Pricing Placement of food/drinks Retail point of sale advertisements	Kilojoule labelling
Implementation barriers/enablers	Reports on barriers and/or enablers of policy implementation	Does not report on or provides extremely limited information on barriers and/or enablers of policy implementation
Population of interest	All	None
Language of publication	English	Not in English

* Policies related to increasing the availability/promotion of healthy food and drinks (i.e., nutrient-rich, fresh or minimally processed foods), or to decreasing the availability/promotion of unhealthy food and drinks (i.e., energy-dense nutrient-poor foods, sugar-sweetened beverages). ** The term “sites” was used to represent scale because the literature on policy implementation retrieved barriers and enablers based on the site and not by outlet. While the number of outlets may have been described in some studies for a given site (for example in schools, one site may have a canteen and two vending machines), implementation was generally by site and the number of outlets in each site was not always described. *** Non-commercial food retail settings, as opposed to commercial food retail settings, refer to food retail within settings whose primary purpose is not to sell food (e.g., hospitals/health services, schools, sports and recreational centres) but to provide food and drinks to staff, students, visitors, and patients.

## Data Availability

Data are contained within the article or Supplementary Material.

## Supplementary Materials

The following are available online at https://www.mdpi.com/article/10.3390/ijerph181910346/s1. Supplementary File S1: Search strategy used for MEDLINE that was adapted for other databases. Supplementary File S2: Summary of included studies. Supplementary File S3: Overarching themes for identified barriers and enablers.
