# Supplementary material for: Implementing Food Environment Policies at Scale: What Helps? What Hinders? A Systematic Review of Barriers and Enablers
Source: Int J Environ Res Public Health. 2021 Sep 30;18(19):10346. doi: 10.3390/ijerph181910346 (PMC8507658; doi:10.3390/ijerph181910346)
Supplement: Supplementary file 1 [file ijerph-18-10346-s001.zip › ijerph-1379647-supplementary/Supplementary File S1_Nguyen et al.pdf]

Supplementary File S1. Search strategy used for MEDLINE that was adapted for other databases.

1. MeSH Health Policy OR health policy.mp
2. MeSH Nutrition Policy OR nutrition policy.mp
3. MeSH Policy OR policy.mp
4. 1, 2 OR 3
5. MeSH Food
6. MeSH Beverages
7. MeSH Energy drinks
8. MeSH Healthy Diet
9. MeSH Sugars
10. MeSH Snacks
11. MeSH Dietary Fats
12. ((food* OR drink* OR beverage*) adj3 (healthy OR unhealthy OR sugar* OR "energy dense" OR "high fat*" OR "low fat" OR sweet* OR processed OR fried OR junk OR "sport* drink" OR energy OR flavo?r*)).mp
13. 5, 6, 7, 8, 9, 10, 11 OR 12
14. MeSH Fund Raising OR Fundrais*.mp
15. MeSH Hospitals OR Hospital*.mp
16. MeSH Health Services OR Health service*.mp
17. MeSH Health Facilities OR Health facilit*.mp
18. Health cent*.mp
19. MeSH Workplace OR Workplace*.mp
20. Aged care.mp
21. MeSH Home Nursing OR Nursing home*.mp
22. MeSH Schools OR School*.mp
23. MeSH Universities OR universit*.mp
24. College*.mp
25. TAFE.mp
26. MeSH Museums OR Museum*.mp
27. Galler*.mp
28. MeSH Animals, Zoo OR Zoo*.mp
29. Sport* facilit*.mp OR sport* club*.mp
30. 14, 15, 16, 17, 18, 19,20, 21, 22, 23, 24, 25, 26, 27, 28 OR 29
31. Implement*.mp
32. Barrier*.mp
33. Enabl*.mp
34. Facilitat*.mp
35. 31, 32, 33, 34 OR 35
36. 4 AND 13 AND 30 AND 35
