# Supplementary material for: Implementing Food Environment Policies at Scale: What Helps? What Hinders? A Systematic Review of Barriers and Enablers
Source: Int J Environ Res Public Health. 2021 Sep 30;18(19):10346. doi: 10.3390/ijerph181910346 (PMC8507658; doi:10.3390/ijerph181910346)
Supplement: Supplementary file 1 [file ijerph-18-10346-s001.zip › ijerph-1379647-supplementary/Supplementary File S2_Nguyen et al.pdf]

Supplementary File S2. Summary of included studies.

Reference, Year, Country	Setting	Policy Description	Scale *	Retail Environment	Measure(s) of Successful/ Unsuccessful Implementation; Time Period of Data Collection	Barriers	How were Barriers Mitigated?	Enablers	Study Quality Rating
Bassler et al., 2013, United States	School	State/regional; district-level policies in 8 districts with strong standards for competitive foods and beverages sold in schools	1	Food and drink supply options in the school environment	Qualitative. Measurement of implementation not described.<br>Semi-structured interviews with 13 district food service directors from eight districts in the United States. Overarching themes for barriers and enablers from interviews with district officers are reported here; 2010-11.	1. Difficulty finding foods and beverages compliant with stronger nutrition standards in the early implementation stages 2. Concerns from district food service directors were also highlighted, such as buy-in from students, staff and parents and financial concerns	Some barriers were mitigated with time (e.g. vendors supplied more compliant foods and beverages)	1. Communication about new standards<br>2. Marketing to promote healthy eating<br>3. Education (e.g. expanding nutrition education curriculum)<br>4. Community engagement<br>5. Leadership and "champions"<br>6. District policy change preceded by changes at the federal/state levels<br>7. Implementing changes over several years<br>8. Implementing changes at the beginning of the year<br>9. School staff modelling healthy eating habits<br>10. Student engagement	High

**Reference, Year, Country**	**Setting**	**Policy Description**	**Scale ***	**Retail Environment**	**Measure(s) of Successful/ Unsuccessful Implementation; Time Period of Data Collection**	**Barriers**	**How were Barriers Mitigated?**	**Enablers**	**Study Quality Rating**
								11. Parental involvement<br>12. Partnering with outside resources<br>13. Improving and increasing participation in school meal program in parallel with competitive food offerings<br>14. Collaborating with vendors	
Budd et al., 2012, United States	School	Federal; 2010 Healthy, Hunger-Free Kids Act (HHFKA) requiring school wellness policy nutrition standards (including for competitive and other foods and beverages)	1	Food and drink supply options in the school environment	Quantitative.<br>Written survey completed by 112 high school administrators (44 principals, 23 nurses, 15 food service/nutrition directors, 11 assistant principals, 10 health/wellness coordinators, 9 others); March-November 2009.	Most cited:<br>1. Lack of time or coordination of policy team (37%)<br>2. Lack of financial resources (33%)<br>3. Not a priority (26%)<br>4. Lack of staff cooperation or support (24%)<br>5. Lack of student acceptance (24%)<br>6. Lack of consequences for noncompliance (20%)	Not described	1. Schools reporting higher quality and effectiveness of implementation reported greater organisational capacity and fewer barriers to implementation<br>2. Schools reporting higher accountability for school wellness policy implementation were more likely to report greater	High

Reference, Year, Country	Setting	Policy Description	Scale *	Retail Environment	Measure(s) of Successful/ Unsuccessful Implementation; Time Period of Data Collection	Barriers	How were Barriers Mitigated?	Enablers	Study Quality Rating
						7. Lack of training, technical assistance, or resources (20%)<br>8. Lack of knowledge or unsure how to proceed (17%)<br>9. Lack of leadership (10%)<br>10. Lack of appropriate food or beverages available from vendors and suppliers (6%)		organisational capacity and fewer barriers	

Reference, Year, Country	Setting	Policy Description	Scale *	Retail Environment	Measure(s) of Successful/ Unsuccessful Implementation; Time Period of Data Collection	Barriers	How were Barriers Mitigated?	Enablers	Study Quality Rating
Dick et al., 2012, Australia	School	State/regional; Smart Choices healthy food and drink supply strategy for Queensland schools; traffic light system; had been mandatory for one school term	1	Tuckshops [a], vending machines, fundraising, sponsorship, advertising	Quantitative.<br>3 concurrent surveys with 991 school principals (online), 607 P&Cs (mailed questionnaire), 513 tuckshop [a] convenors (CATI) from Queensland primary and secondary state schools; May-July 2007.	1. School location. Urban school principals more likely than rural ones to report overall implementation as excellent/good (87% vs 79%, P≤0.001); urban school P&Cs more likely to report increased tuckshop [a] profits (19% vs 10%, P≤0.01); urban school tuckshop [a] convenors more likely to strongly agree/agree that they had reliable access to healthier products (86% vs 69%, P≤0.001) and to report increased healthy food and drink availability (refer to paper for detailed results for particular foods/water); urban P&Cs more likely to attend information session (43% vs. 30%, P≤0.001) and urban tuckshop [a] convenors too (52% vs 37%, P≤0.001).<br>2. Primary or secondary school. Primary school	Not described; suggested future actions	1. Good understanding of Smart Choices (79% principals, 86% P&Cs, 89% tuckshop [a] convenors), classification (≥95% of P&Cs and tuckshop [a] convenors) and confidence in implementing Smart Choices (99% tuckshop [a] convenors)<br>2. High engagement and coordination from principals and P&Cs. 91% principals and 86% P&Cs agreed that Smart Choices is an important strategy to improve children's health. Arrangements to limit red products supply: 97% principals, 93% P&Cs<br>3. Support from school community (64% principals, 58% P&Cs)<br>4. Financial viability. Belief in healthy school	High

Reference, Year, Country	Setting	Policy Description	Scale *	Retail Environment	Measure(s) of Successful/ Unsuccessful Implementation; Time Period of Data Collection	Barriers	How were Barriers Mitigated?	Enablers	Study Quality Rating
						principals more likely than secondary school principals to report implementation in curriculum activities (98% vs 95%, P≤0.05) and school excursions (95% vs 91%, P≤0.05); secondary schools P&Cs more likely to report decreased tuckshop[a] profits (47% vs 26%, P≤0.01); primary school tuckshop[a] convenors more likely to strongly agree/agree that they were satisfied with the range of green and amber products available (82% vs 70%, P≤0.01) and to report increased availability of fruit on their menus (83% vs 67%, P≤0.01); secondary school principals more likely to report excellent/good understanding of Smart Choices (85% vs 77%, P≤0.05); secondary school convenors more likely to		tuckshop[a] (78% P&Cs) and fundraising (62%) viability. P&Cs reported increased (15%) or unchanged (41%) tuckshop[a] profits.	

Reference, Year, Country	Setting	Policy Description	Scale *	Retail Environment	Measure(s) of Successful/ Unsuccessful Implementation; Time Period of Data Collection	Barriers	How were Barriers Mitigated?	Enablers	Study Quality Rating
						report attending training and networking opportunities (P-value not provided).			

Reference, Year, Country	Setting	Policy Description	Scale *	Retail Environment	Measure(s) of Successful/ Unsuccessful Implementation; Time Period of Data Collection	Barriers	How were Barriers Mitigated?	Enablers	Study Quality Rating
Downs et al., 2012, Canada	School	State/regional; Alberta Nutrition Guidelines for Children and Youth (ANGCY); voluntary; released in 2008	1	Food and drink supply options in the school environment	Mixed methods. Telephone survey (357 schools, 64% participation rate) with school principals (90%), teachers (4%) and other key school staff (6%); September-December 2013	Barriers for adopters: Parents' resistance to change, cost of healthy foods, lack of knowledge, student preferences, physical location of the school (close proximity to convenience stores and fast food outlets), logistics relating to healthy food provision. The most frequently cited barriers differed based on innovation characteristic considered. Regardless of school's geographical location, school size or presence of a school champion, parents' resistance to change was one of the most cited barriers.	Not described; suggested future actions	Not described	High

Reference, Year, Country	Setting	Policy Description	Scale *	Retail Environment	Measure(s) of Successful/ Unsuccessful Implementation; Time Period of Data Collection	Barriers	How were Barriers Mitigated?	Enablers	Study Quality Rating
Girona et al., 2018, Uruguay	School	Federal decree in 2014; healthy snacking initiative	1	Food and drink supply in secondary schools (cafeterias, tuckshops[a], vending machines, restaurant)	Mixed methods.<br>Semi-structured interviews with school principals from 38 primary schools (24 public, 14 private) and 21 secondary schools (8 public and 13 private) and on-site survey of foods and beverages sold/advertised in schools; July-November 2016.	Law characteristics:<br>1. Lack of communication from authorities and lack of knowledge about the content of the initiative and its requirements<br>2. Difficulty identifying products<br>3. Lack of legal sanctions<br>School characteristics:<br>1. Primary vs secondary school<br>2. Public vs private<br>3. Lack of interest from school principals<br>4. Lack of facility in secondary schools to prepare homemade food<br>External factors:<br>1. Children and adolescent preferences for unhealthy products<br>2. Decreased profits if secondary school students purchased unhealthy foods from stores outside school<br>3. Advertising	Not described	Law characteristics:<br>1.Control/inspection from the authorities<br>School characteristics:<br>1. Interest from school principals	High

Reference, Year, Country	Setting	Policy Description	Scale *	Retail Environment	Measure(s) of Successful/ Unsuccessful Implementation; Time Period of Data Collection	Barriers	How were Barriers Mitigated?	Enablers	Study Quality Rating
						4. Family habits			

Reference, Year, Country	Setting	Policy Description	Scale *	Retail Environment	Measure(s) of Successful/ Unsuccessful Implementation; Time Period of Data Collection	Barriers	How were Barriers Mitigated?	Enablers	Study Quality Rating
Masse et al., 2013, Canada	School	State/regional; Food and Beverage Sales in Schools (FBSS) guidelines; full implementation mandatory since 2007-08	2	Food and drink supply options in the school environment. Examples: cafeterias, vending machines, school stores, classroom rewards, fundraising	Qualitative. Semi-structured interviews with 50 school informants (17 principals, 33 teacher/school informants) from 17 schools (10 elementary, 1 junior high, 1 senior high, 5 high schools) in British Columbia, Canada; 2010-11.	1. Revenue loss<br>2. Scope of guidelines unclear (which type of food provision, when and where guidelines applied)<br>3. Difficulty finding suitable fundraising alternatives<br>4. Parents' perceptions that educators are overstepping boundaries<br>5. Schools also dealing with food insecurity	Not described	1. Welcomed change<br>2. Compatible with schools'/teachers' expectations<br>3. Provincial resources to support implementation<br>4. Able to consult nutritionist<br>5. Local suppliers that complied with guidelines<br>6. Top-down approach	High
Matthews et al., 2011, United Kingdom	School	Federal legislation in 2007; food standards for schools (New Standards)	1	Food and drink vending machine supply in secondary school setting	Mixed methods. Based on postal (279 schools at baseline, 231 at year 3) and visit-based inventory (62 schools at baseline, 29 schools at year 3) surveys and semi-structured interviews (29 inventory schools); mostly catering managers; three time periods (2006-07, 2007-08 and 2008-09).	1. Misunderstanding of suitable vended products<br>2. Unavailability of compliant vended products<br>3. Commercial viability<br>4. Loss of vending machine income<br>5. Non-compliance of sixth form vending due to school structures and hierarchies<br>6. Perceived as nanny state approach<br>7. Resistance to the New Standards	Vending machine suppliers' product lists	1. Availability of compliant vended products<br>2. Whole school approach	High

Reference, Year, Country	Setting	Policy Description	Scale *	Retail Environment	Measure(s) of Successful/ Unsuccessful Implementation; Time Period of Data Collection	Barriers	How were Barriers Mitigated?	Enablers	Study Quality Rating
						8. Students purchasing prohibited foods externally			
Miller et al, 2015, Australia	Hospital/ health facility	State/regional; A Better Choice Healthy Food and Drink Supply Strategy for Queensland Health Facilities; traffic light system; mandatory since September 2008	1	Food retail outlets, vending machines, fundraising	Mixed methods. Online survey (134 facility managers) and telephone interviews (24 A Better Choice district contact officers); May 2009	60.3% reported barriers:<br>1. Perceived customer dissatisfaction (41%)<br>2. Difficulty accessing suitable green category products (23.1%)<br>3. Perceived lack of demand for healthy foods and drinks (20.9%)<br>4. Concern over loss of profit (11.9%)<br>5. Lack of management support (3.7%)	Not described; suggested future actions	1. Staff found promotional posters very/somewhat useful (70% of facility managers)<br>2. Toolkit (56.3% of facility managers), strategy document (54.3%), brochures (50.9%) and website (47%) found to be very/somewhat useful	High

Reference, Year, Country	Setting	Policy Description	Scale *	Retail Environment	Measure(s) of Successful/ Unsuccessful Implementation; Time Period of Data Collection	Barriers	How were Barriers Mitigated?	Enablers	Study Quality Rating
Moran et al., 2016, United States	Hospital/ health facility	Local (city); Healthy Hospital Food Initiative (HHFI) from the New York City Department of Health and Mental Hygiene; 2010; voluntary. Private hospitals joined by committing to implement 4 sets of standards (patient meals, beverage vending, food vending, cafeterias/cafes). Public hospitals already implemented the first 3 standards and joined by voluntarily implementing the standards for cafeterias/cafes.	1	Cafeterias, cafes, beverage/food vending machines	Quantitative. Hospitals required to meet ≥75% of criteria within each set of standards. Communication with hospital staff, on site inventory, diagrams of products stocked in vending machines, menu analyses; 40 hospitals (16 public, 24 private); 28 hospital cafeterias and cafes; number of vending machines not reported; 2010-2014	1. Vending machines managed by outside vending machine suppliers with limited product availability meeting nutrient criteria 2. Several cafeterias were franchisees of a larger chain making local changes difficult 3. Limited data on nutrient content of foods/meals bought from the cafeterias requiring high time investment from staff and health department dietitians	Increasing demand on vending machine supplier to provide products meeting requirements	1. HHFI part of multi-sector effort (workplaces, childcare, food retailers) to improve healthy food and drink supply in the city 2. Support from high-level officials/leaders 3. Comprehensive framework: implementation guides, promotional resources, technical assistance from 2 full-time dietitians, hospital communications with employees, monthly progress reports 4. Public recognition of accomplishments	Mod

Reference, Year, Country	Setting	Policy Description	Scale *	Retail Environment	Measure(s) of Successful/ Unsuccessful Implementation; Time Period of Data Collection	Barriers	How were Barriers Mitigated?	Enablers	Study Quality Rating
Olstad et al., 2011, Canada	Sport/ recreation	State/regional; ANGCY; voluntary; released in 2008	1	Food and drink supply options in recreational settings	Mixed methods. Telephone survey with managers of 141 publicly funded recreational facilities one year after ANGCY release; June-December 2009	Some managers did not identify any barriers.<br>Attributes of ANCGY:<br>1. Perceived incompatibility of the guidelines with customer demands<br>2. Not as profitable<br>3. Increased complexity, less convenient<br>4. Knowing that children bought unhealthy foods from nearby convenience stores<br>5. Unlikely to observe the benefits<br>6. Changes made could have been minor	Not described; suggested future actions	ANCGY champion	High

Reference, Year, Country	Setting	Policy Description	Scale *	Retail Environment	Measure(s) of Successful/ Unsuccessful Implementation; Time Period of Data Collection	Barriers	How were Barriers Mitigated?	Enablers	Study Quality Rating
Orava et al., 2017, Canada	School	State/regional; Ontario School Food and Beverage Policy; traffic light system; mandatory since 2011	2	Food and drink supply options in the school environment. Examples: cafeterias, vending machines, tuckshops[a], bake sales, sport events	Mixed methods. Written survey and onsite school food environmental scan checklist (n=25 [8 elementary, 17 secondary] school representatives that are knowledgeable about healthy eating-related initiatives at their schools); 2012-13 and 2014.	1. School staff members not consulted often on discussions related to healthy eating<br>2. Forming partnerships can be challenging<br>3. Lack of funding	To increase stakeholder engagement by involving community members in discussions and supporting existing partnerships with external organisations	1. School health champions (e.g. teachers running/ supervising programs during non-classroom time)<br>2. Need for multiple representations from positions of power (e.g. Ministry of Education, school board, school principal, school champion) to work with school stakeholders	High
Pettigrew et al., 2012, Australia	School	State/regional; Western Australia Healthy Food and Drink Policy; traffic light system; mandatory since 2007	1	Food and drink supply options in the school environment	Mixed methods. Semi-structured interviews with 10 school principals and online survey with 310 principals (44% response rate, 75% primary school, 60% metropolitan), Post implementation period (specific time period unclear).	From interviews:<br>1. Complaints from parents and children<br>2. Resistance from canteen staff<br>3. Increasing food costs	Complaints were quickly resolved given the non-negotiable nature of the policy. Training for canteen staff was helpful with development and delivery of appropriate	From interviews:<br>1. General parental awareness of problem of childhood obesity and appreciation for schools' efforts<br>2. Policy providing legitimacy and authority to make changes<br>3. Key stakeholders informed throughout implementation process (e.g. information	High

Reference, Year, Country	Setting	Policy Description	Scale *	Retail Environment	Measure(s) of Successful/ Unsuccessful Implementation; Time Period of Data Collection	Barriers	How were Barriers Mitigated?	Enablers	Study Quality Rating
							menus, and it took into account infrastructure and staffing constraints.	materials provided by the Department of Education, school communication with parents and the community)	
Pettigrew et al., 2013, Australia	School	State/regional; Western Australia Healthy Food and Drink Policy; traffic light system; mandatory since 2007	1	Food and drink supply options in the school environment	Mixed methods.<br>Six semi-structured focus groups (32 parents) and 48 school stakeholder interviews (10 principals, 18 teachers, 10 canteen managers, 10 presidents of P&C committees); 12-18 months following policy introduction. Telephone questionnaires with 1152 parents (September 2008) and internet-based questionnaires with 263 principals from primary and secondary schools	From qualitative analysis:<br>1. Parents concerned about reduced parental autonomy to make choices for their children	Not described; suggested future actions	From qualitative analysis:<br>1. Parental support for the policy<br>2. Understanding of traffic light system<br>3. Adequate support materials<br>From quantitative analysis, variables most strongly related to being fully compliant:<br>1. Canteen being healthy prior to the policy<br>2. P&Cs approving menu changes	High

Reference, Year, Country	Setting	Policy Description	Scale *	Retail Environment	Measure(s) of Successful/ Unsuccessful Implementation; Time Period of Data Collection	Barriers	How were Barriers Mitigated?	Enablers	Study Quality Rating
					(September 2008-February 2009).			3. Ease of policy implementation<br>4. Kitchen setup<br>5. Prior involvement with a voluntary food categorisation system<br>6. Training and assistance provided by the Department of Education for canteen managers<br>7. Policy not requiring major changes to the menu<br>8. Policy reflecting parents' views<br>9. Policy not ignoring parents' rights to choose types of food for their children<br>10. Attitude of parents	
Pettigrew et al., 2018, Australia	School	State/regional; Western Australia Healthy Food and Drink Policy; traffic light system; mandatory since 2007	1	Food and drink supply options in the school environment	Mixed methods. Online survey (n=307) involving multiple stakeholders (principals, teachers, canteen managers, P&C presidents); in 2008 (1 year	% agreement from all stakeholders for the following perceived policy outcomes:<br>1. Children have shown interest in the traffic light	Not described; suggested future actions	% agreement from all stakeholders for the following perceived policy outcomes:<br>1. Policy is a good opportunity to teach children about healthy	High

Reference, Year, Country	Setting	Policy Description	Scale *	Retail Environment	Measure(s) of Successful/ Unsuccessful Implementation; Time Period of Data Collection	Barriers	How were Barriers Mitigated?	Enablers	Study Quality Rating
					post implementation) and 2016 (10 year post implementation).	system (35% in 2008, 42% in 2016)<br>2. Policy did not reflect parents' views on children's diets (69% in 2008, 63% in 2016)		eating (79% in 2008, 90% in 2016)<br>2. Traffic light system is easy to understand (77% in 2008, 88% in 2016)<br>3. Policy has been effective in making foods provided at school healthier (84% in 2008, 85% in 2016)<br>4. It has not been difficult to implement the policy at our school (81% in 2008, 87% in 2016)	
Reeve et al., 2018, Philippines	School	Federal; Department of Education Order no 8 (2007)	2	Food and drink supply options in the school environment (example: canteens)	Qualitative.<br>Semi-structured interviews with 21 policymakers and stakeholders across the Greater Manila region involved in school food policy making and implementation; May 2016.	1. Lack of human and financial resources dedicated to implementation<br>2. Limited capacity building<br>3. Not a top priority for principals<br>4. Lack of implementation monitoring and enforcement	Not described	Not described	High

Reference, Year, Country	Setting	Policy Description	Scale *	Retail Environment	Measure(s) of Successful/ Unsuccessful Implementation; Time Period of Data Collection	Barriers	How were Barriers Mitigated?	Enablers	Study Quality Rating
						5. Conflicts of interest with large food companies			
Roberts et al., 2009, United States	School	State/regional; Texas School Nutrition Policy; mandatory since 2004	2	Food and drink supply options in the school environment. Examples: cafeterias, vending machines, fundraising	Qualitative. Semi-structured telephone interviews with 24 school principals and 10 school food service directors from 24 middle schools from 10 Texas Education regions; 2005.	1. Initial resistance to the policy (e.g. from students, teachers)<br>2. Lack of communication and involvement with key people affected (e.g. parents, teachers, principals, food service directors)<br>3. Government role (top-down approach)<br>4. Lower revenue from vending machines and fundraising	Suggested future actions	1. Students' acceptance of the policy	High
Schuler et al., 2018, United States	School	Federal; 2010 HHFKA and 2016 final rule requiring school to meet expanded local wellness policies requirements	1	Food and drink supply options in the school environment	Mixed methods. Quantitative surveys (744 schools, 62% elementary) and semi-structured telephone interviews (n=20 random subsample; 60%	Common to both quantitative and qualitative methodologies:<br>1. Staff (lack of buy in and commitment)<br>2. Time/competing priorities	Not described	Common to both quantitative and qualitative methodologies: staff (supportive). Additional from qualitative analysis: funding.	High

Reference, Year, Country	Setting	Policy Description	Scale *	Retail Environment	Measure(s) of Successful/ Unsuccessful Implementation; Time Period of Data Collection	Barriers	How were Barriers Mitigated?	Enablers	Study Quality Rating
					elementary) completed mostly by principals/assistant principals. Time period unclear.	3. Funding from school system<br>4. Parents/families<br>Additional from qualitative analysis:<br>Food service (communication, unhealthy offerings).<br>Top 5 most common based on quantitative analysis:<br>1. Parents/families<br>2. Federal/state regulations<br>3. Students<br>4. Community<br>5. School system		Top 5 most common based on quantitative analysis:<br>1. School system<br>2. Teachers<br>3. Food service<br>4. Federal/state regulations<br>5. Designated coordination person	
Vine et al., 2013, Canada	School	State/regional; Ontario School Food and Beverage Policy in 2011 encompassing nutritional standards	2	Food and drink supply in the school environment	Qualitative. Measures of implementation not described; in person and telephone interviews with 22 key school stakeholders (8 community-level, 14 school-level: secondary school principals, vice principals, teachers, administrators) to assess barriers/	Economic environment:<br>1. Cost of healthy food for sale (more of a concern for schools with low-income students)<br>2. Decreased revenue<br>Physical environment:<br>1. Proximity to external food outlets<br>Political environment:<br>1. Restrictive policy guidelines	Not described	Sociocultural environment:<br>1. Buy-in from key school-level stakeholders and school champions	High

Reference, Year, Country	Setting	Policy Description	Scale *	Retail Environment	Measure(s) of Successful/ Unsuccessful Implementation; Time Period of Data Collection	Barriers	How were Barriers Mitigated?	Enablers	Study Quality Rating
					enablers; December 2011 - March 2012.	2. Clarifying role of key stakeholders in policy implementation			
Woodward-Lopez et al., 2010, United States	School	State/regional; Californian legislation relating to nutrition standards for competitive foods (Senate Bill 12) and beverages (Senate Bill 965); mandatory since July 2007	1	Food and drink supply options in the school environment	Quantitative.<br>School site visits (99 schools) to assess pre-post legislation changes in school food and beverage offerings and degree of compliance with nutrition standards; barriers to implementation assessed by interactive PDF survey completed by food service directors/ supervisors in one study ("study 1"; 56 high schools) and on-site school wellness team interview in	From study 1:<br>1. Costs of foods and beverages (61%)<br>2. Labour costs (48%)<br>3. Student preferences (21%)<br>4. Student opposition to change (7%)<br>5. Lack of district/school support (<10%)<br>From study 2:<br>1. Lack of buy in from school personnel benefiting financially from competitive food and beverage sale.<br>2. Competing school priorities.	Not described	Not described	Mod

**Reference, Year, Country**	**Setting**	**Policy Description**	**Scale ***	**Retail Environment**	**Measure(s) of Successful/ Unsuccessful Implementation; Time Period of Data Collection**	**Barriers**	**How were Barriers Mitigated?**	**Enablers**	**Study Quality Rating**
					another study ("study 2"; 8 elementary, 8 middle and 8 high schools); 2004-09.				

Abbreviations: ANGCY=Alberta Nutrition Guidelines for Children and Youth; CATI=Computer-assisted telephone interview, Mod=moderate, P&Cs=Parent & Citizens' Associations. *1=large scale (>25 sites), 2=not so large scale (≥10 and ≤25 sites). [a] A tuckshop is a food shop within or close to the school.
