# Supplementary material for: Implementing Food Environment Policies at Scale: What Helps? What Hinders? A Systematic Review of Barriers and Enablers
Source: Int J Environ Res Public Health. 2021 Sep 30;18(19):10346. doi: 10.3390/ijerph181910346 (PMC8507658; doi:10.3390/ijerph181910346)
Supplement: Supplementary file 1 [file ijerph-18-10346-s001.zip › ijerph-1379647-supplementary/Supplementary File S3_Nguyen et al.pdf]

Supplementary File S3. Overarching themes for identified barriers and enablers

**Overarching themes for identified barriers and enablers**
Perceptions of the policy
• Understanding/clarity<br>• Alignment with stakeholders' views/demands<br>• Nanny state/top down approach<br>• Influencing parental autonomy<br>• Restrictive nature
Organisational and contextual factors
Organisational
• Time, money, staff, resources<br>• Leadership, school/policy champion, management commitment, organisational capacity<br>• Stakeholder engagement, whole school approach, prioritisation<br>• Implementation processes adopted
Contextual
• Supply of policy-compliant/healthy products<br>• Supply of affordable products<br>• Marketing influences<br>• School or facility location (e.g. rural, urban), type, history, structures<br>• Information, guidance and/or training support from the policy level or higher level support<br>• Monitoring/enforcement from the policy level<br>• External partnerships<br>• Part of a multisector effort
Stakeholder responses
• Resistance to change (e.g. personal preferences, family habits) /acceptance of change (e.g. positive attitude)<br>• Complaints<br>• Going to buy foods/drinks externally<br>• Public recognition of accomplishments<br>• Ease of implementation, policy providing legitimacy to make changes
Impacts
• Profits/revenue/commercial viability<br>• Food cost/food insecurity<br>• Labour cost<br>• Fundraising alternatives<br>• Food offerings<br>• Purchase displacement
